# Supplementary figures and images for: Prefrontal cortical activity predicts the occurrence of nonlocal hippocampal representations during spatial navigation
Source: PLoS Biol. 2021 Sep 16;19(9):e3001393. doi: 10.1371/journal.pbio.3001393 (PMC8494358; doi:10.1371/journal.pbio.3001393)

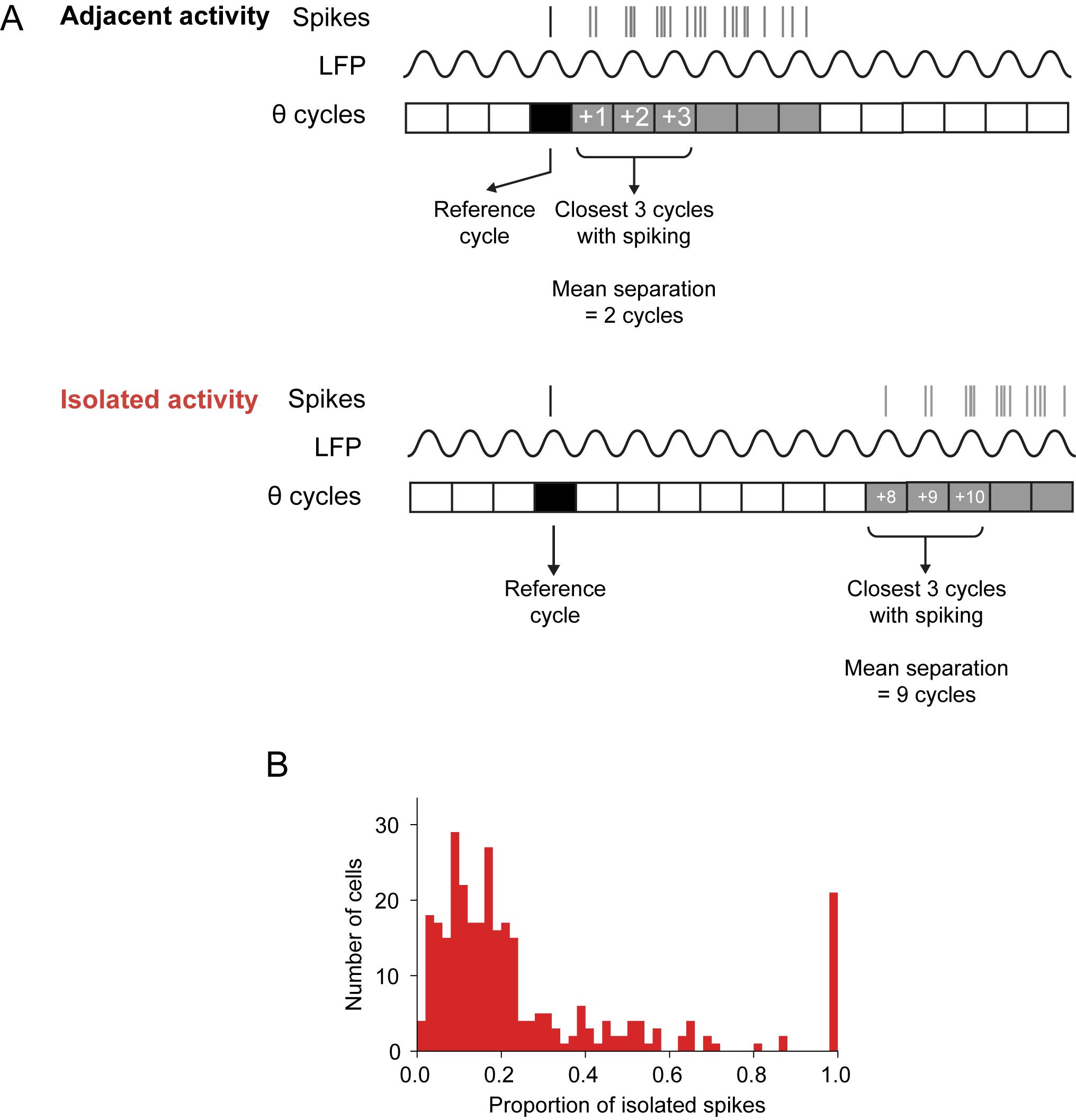

Supplement: S1 Fig — (A) Classification of adjacent versus isolated activity based on temporal separation between theta cycles with spiking. (B) Proportion of spikes (adjacent and isolated) classified as isolated for all CA1 cells. Median: 0.17 ± 0.018 (95% CI). (TIF) [file pbio.3001393.s001.tif]

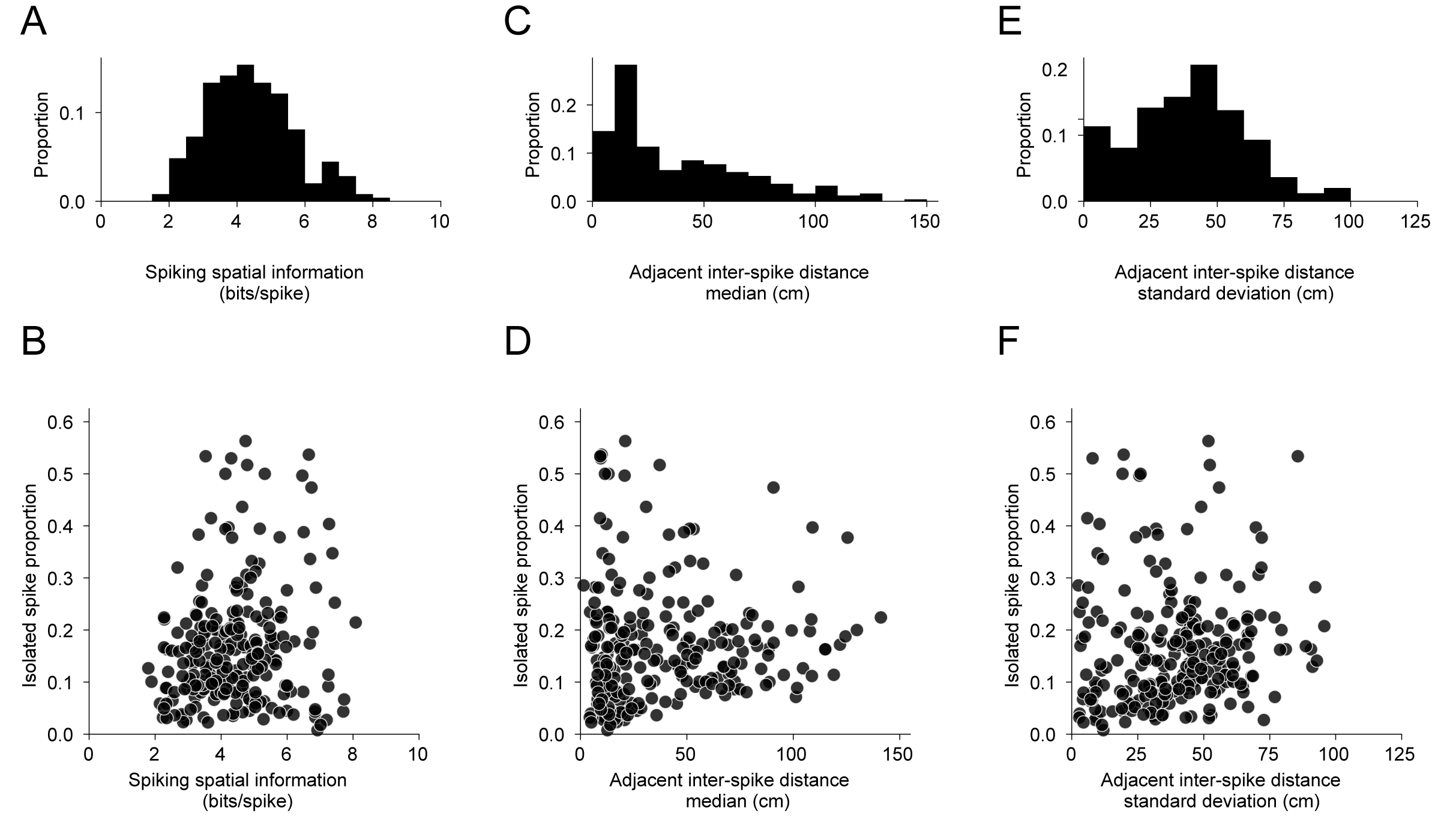

Supplement: S2 Fig — (A) Distribution of spatial information for CA1 cells (n = 247). Median: 4.35 bits/spike. (B) The proportion of isolated spikes is very weakly correlated with spiking spatial information (n = 247). R2 = 0.0246 p = 0.014. (C) Distribution of the median distance between adjacent spikes (n = 247). Median: 24.04 cm. (D) The proportion of isolated spikes is not significantly correlated with the median distance between locations at which adjacent spikes were observed (n = 247). R2 = 0.0081 p = 0.16. (E) Distribution of the standard deviation of distances between locations at which adjacent spikes were observed (n = 247). Median: 40.18 cm. (F) The proportion of isolated spikes is not significantly correlated with the standard deviation of distances between locations at which adjacent spikes were observed (n = 247). R2 = 0.0089 p = 0.14. (TIF) [file pbio.3001393.s002.tif]

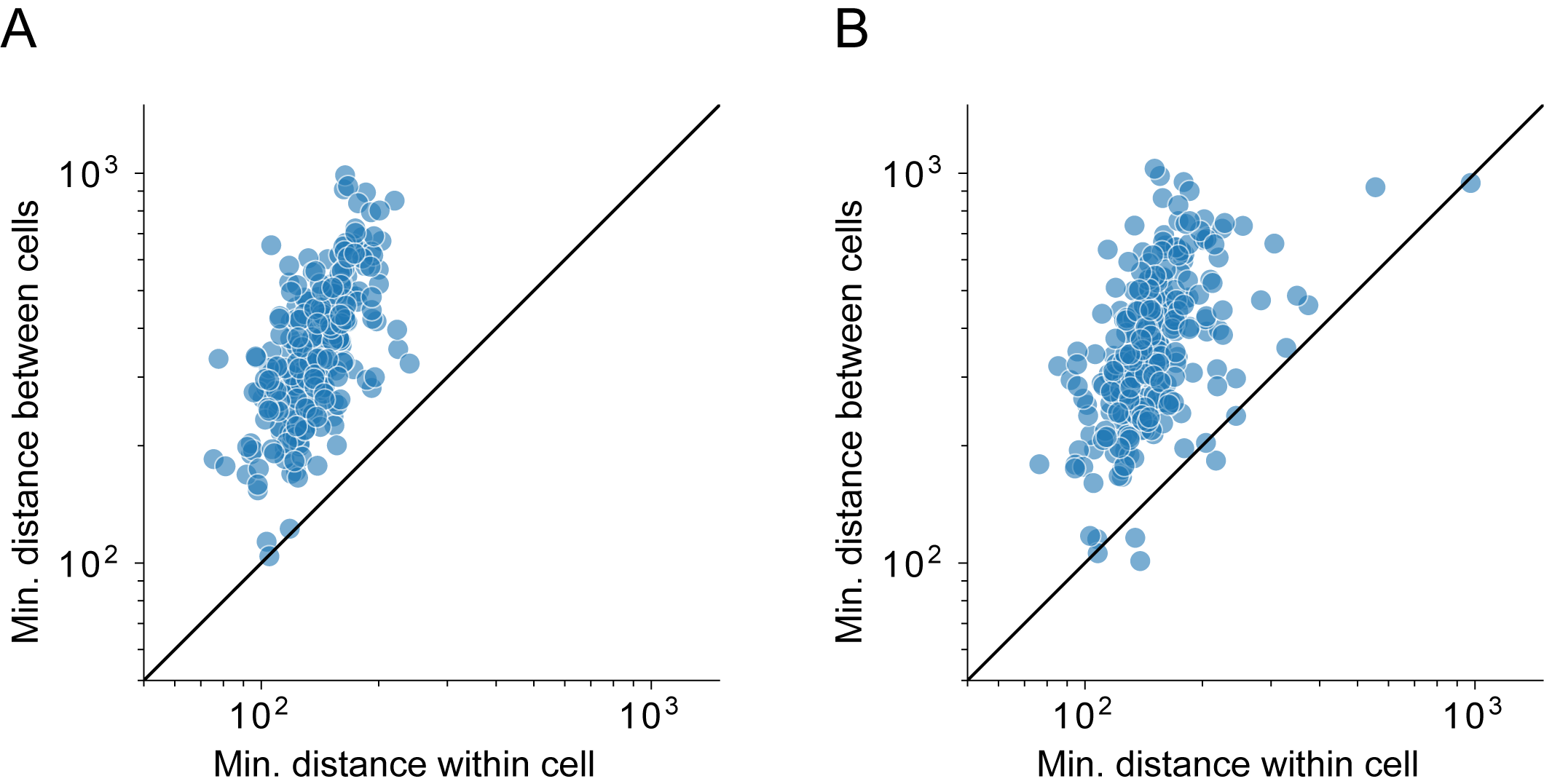

Supplement: S3 Fig — (A) Minimum Euclidean distance between the spike waveform of adjacent activity spikes within each cell versus all other cells recorded on the same tetrode (n = 260). Wilcoxon signed rank test: p = 2.9 × 10^−51^. (B) Minimum Euclidean distance between the spike waveforms of spikes classified as isolated activity within each cell versus all other cells recorded on the same tetrode (n = 276). Wilcoxon signed rank test: p = 8.8 × 10^−51^. (TIF) [file pbio.3001393.s003.tif]

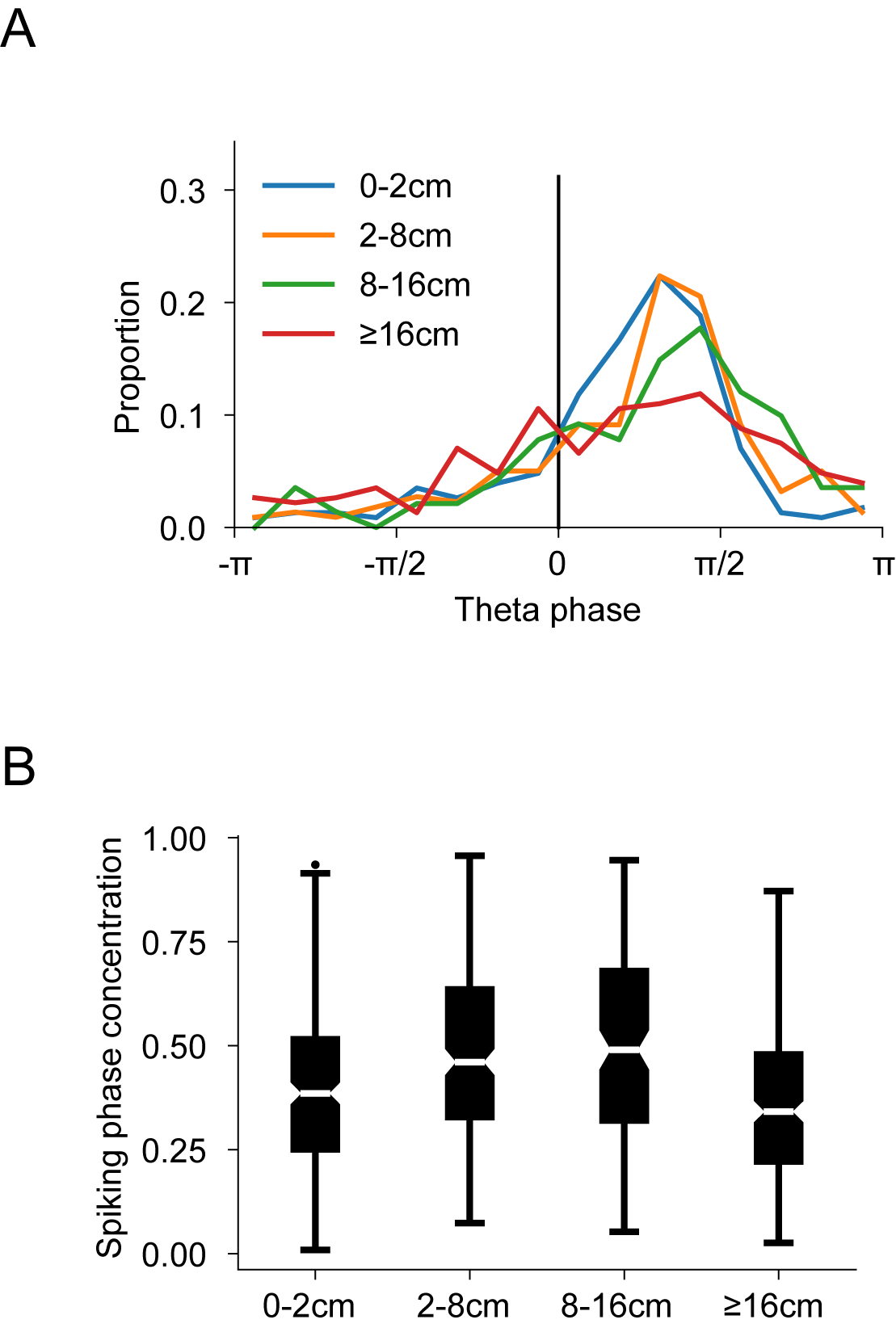

Supplement: S4 Fig — (A) Mean theta phase preference distribution for isolated spiking grouped by distance to its nearest 10 adjacent spikes. Mean phase: 0.80, 0.97, 1.13, and 0.89 for distances 0–2, 2–8, 8–16, and ≥16 cm, respectively. Only cells with 5 or more spikes are included for each distance category. Kruskal–Wallis test: H (3, 815) = 9.79, p = 0.020. (B) Mean spiking phase concentration for isolated spiking grouped by distance to its nearest 10 adjacent spikes. Median phase concentration: 0.39, 0.46, 0.49, and 0.34 for distances 0–2, 2–8, 8–16, and ≥16 cm, respectively. Only cells with 5 or more spikes are included for each distance category. Kruskal–Wallis test: H (3, 815) = 52.8, p = 2.03 × 10^−11^. (TIF) [file pbio.3001393.s004.tif]

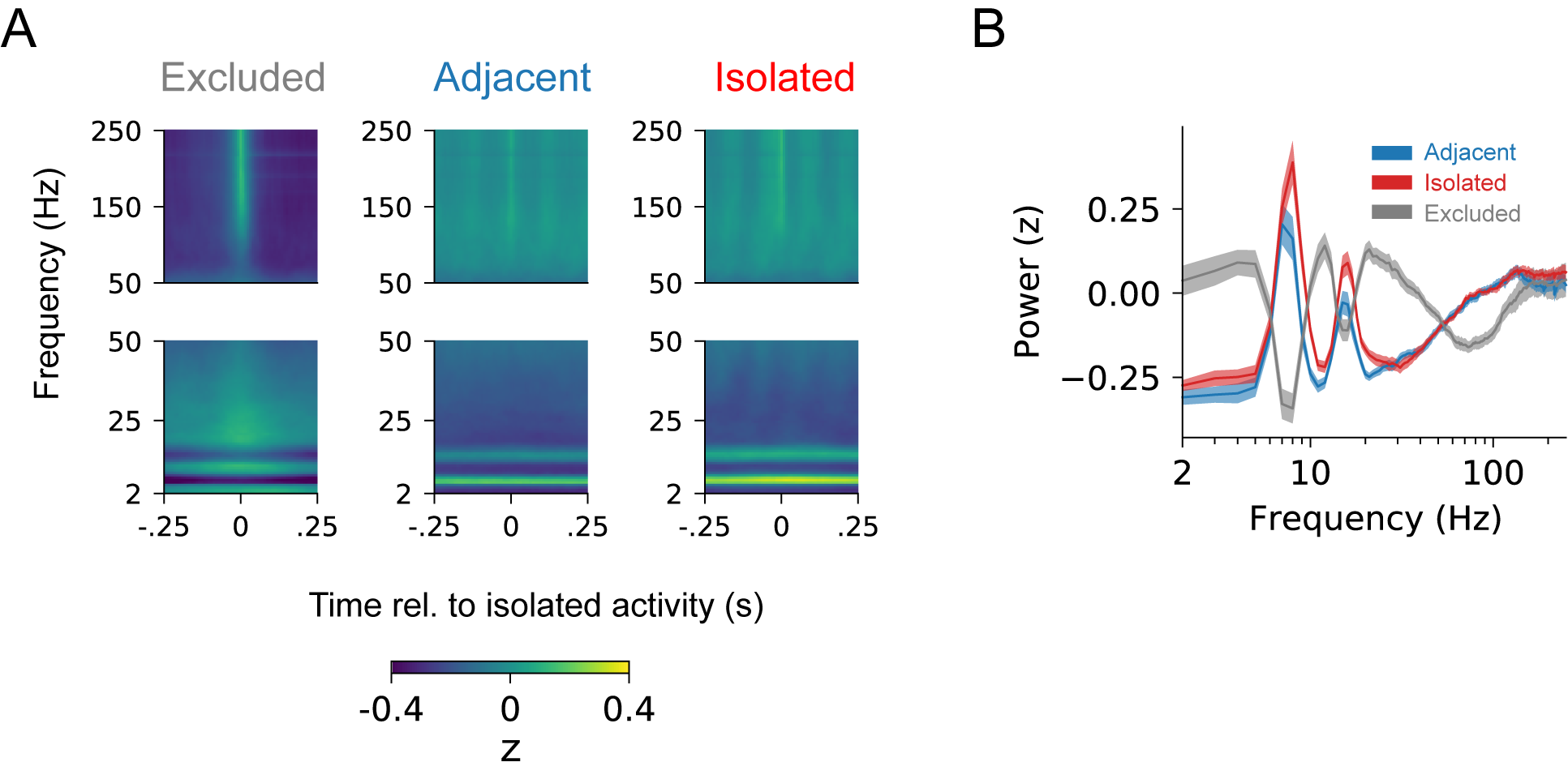

Supplement: S5 Fig — (A) Mean spike triggered spectrogram for excluded (left), adjacent (center), and isolated (right) spiking activity (n = 170 cells). Top panels show frequency ranges 50 to 250 Hz. Bottom panels show frequency ranges 2 to 50 Hz. (B) Mean spectral power for a 50-ms window centered at 0-ms lag (median ± 95% CI). (TIF) [file pbio.3001393.s005.tif]

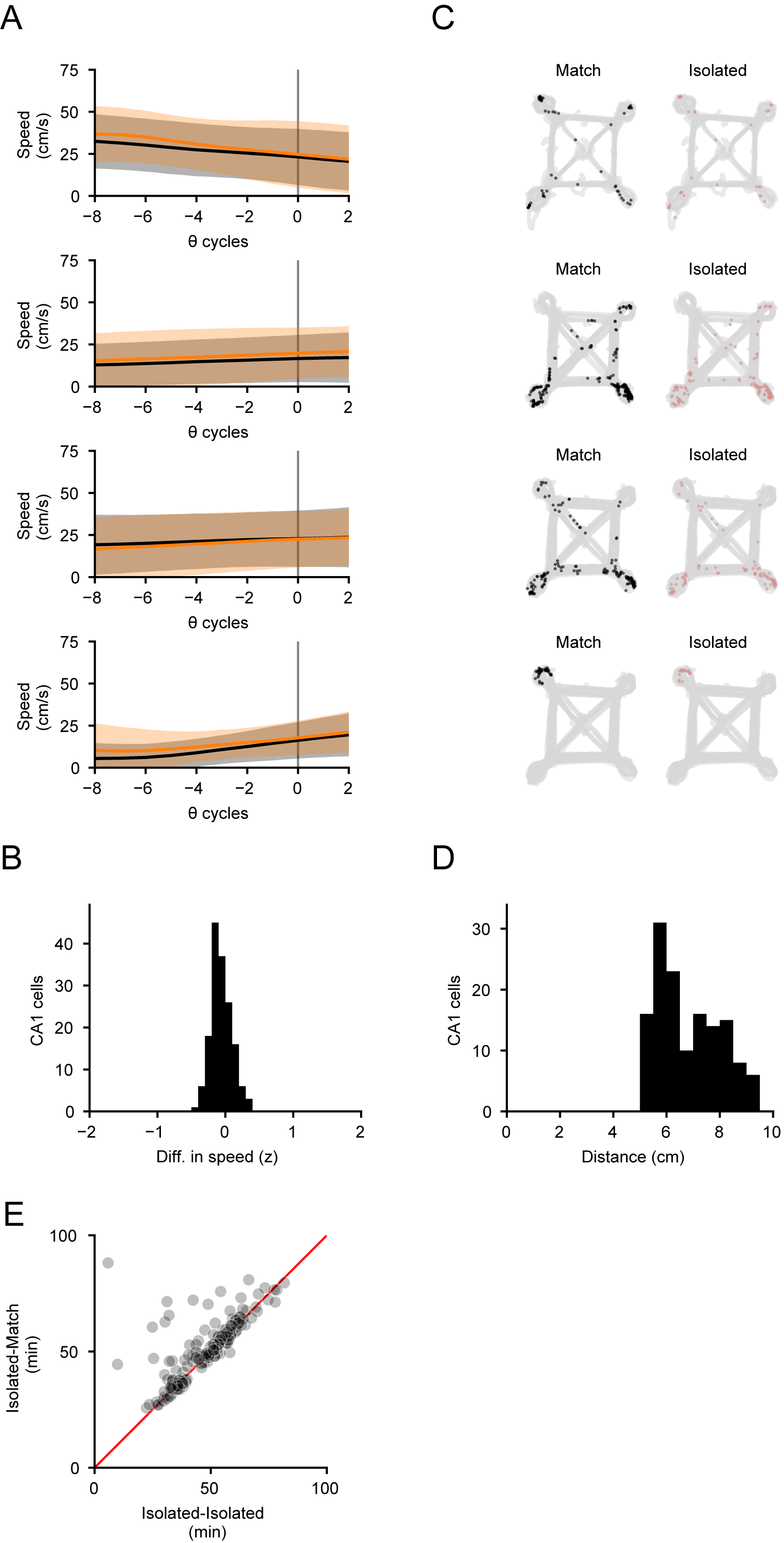

Supplement: S6 Fig — (A) Speed match profiles for examples in Fig 6B. (B) Distribution of mean difference in speed between matched and isolated cycles. Speed profiles of matched cycles were on average within −0.06 standard deviations of the speed profile of the isolated cycles. The difference is expressed as a z-score normalized against the speed distribution of isolated cycles. (C) Location match profiles for examples in Fig 6B. (D) Distribution of the mean distance in cm between matched and isolated cycles. The location of the animal on matched cycles was on average 7.5 cm from the location of the isolated cycle. (E) Intercycle time interval between isolated cycles or isolated and matched cycles for CA1 cells with at least 100 cycles (n = 158). Sign test p = 0.0669. (TIF) [file pbio.3001393.s006.tif]

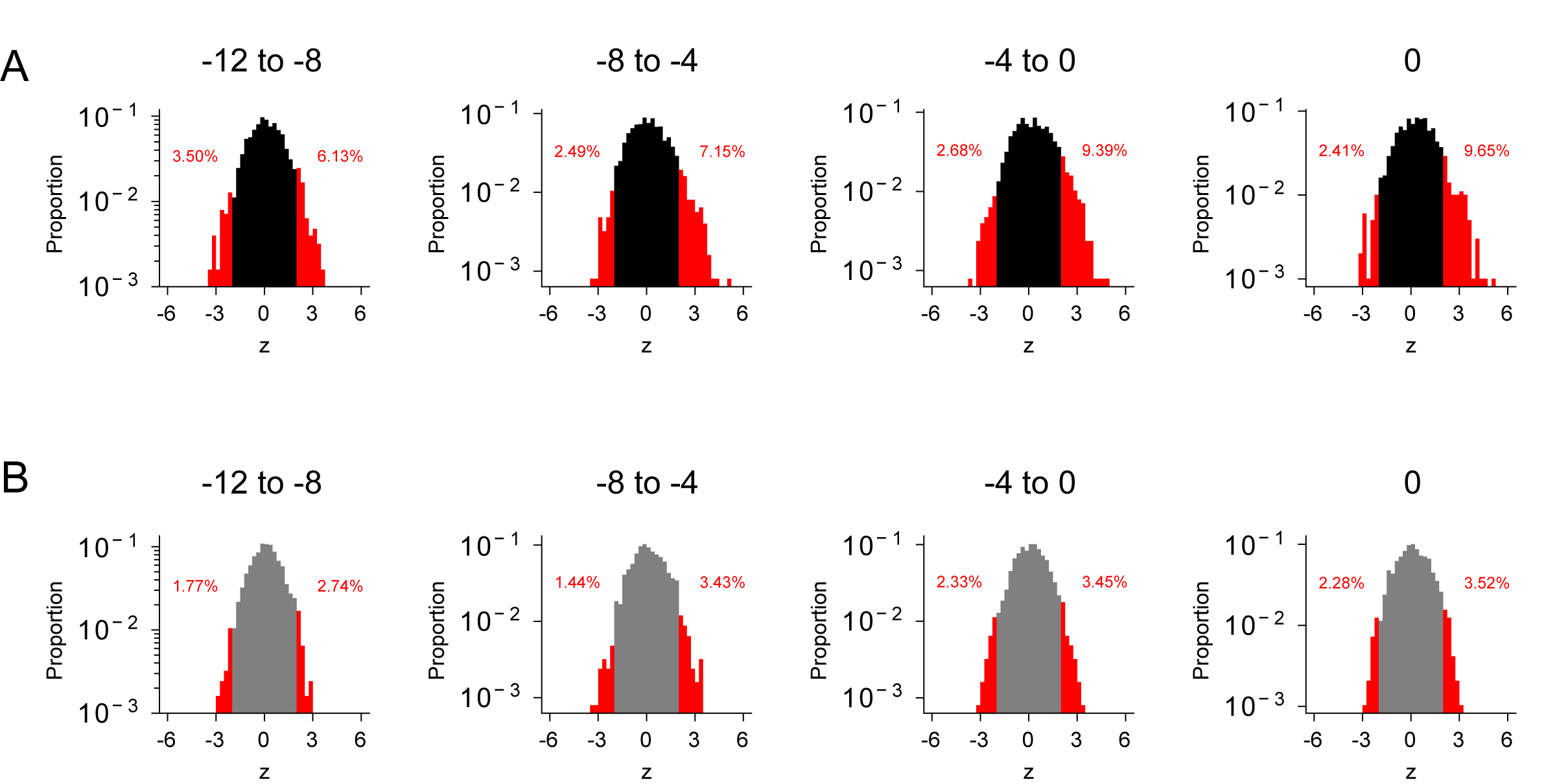

Supplement: S7 Fig — (A) Distribution of PFC firing rate change (z) for time windows before and during CA1 isolated spiking in Fig 6C. Bins above and below 2 z are colored red and the proportion of the distribution are printed in red. Only PFC–CA1 cell pairs where PFC spiking occurs in at least 25% of isolated or matched cycles are included. This selects for PFC cells with higher spiking rates. (B) Distribution of permuted PFC firing rate change (z) for time windows before and during CA1 isolated spiking in Fig 6C. PFC, prefrontal cortex. (TIF) [file pbio.3001393.s007.tif]

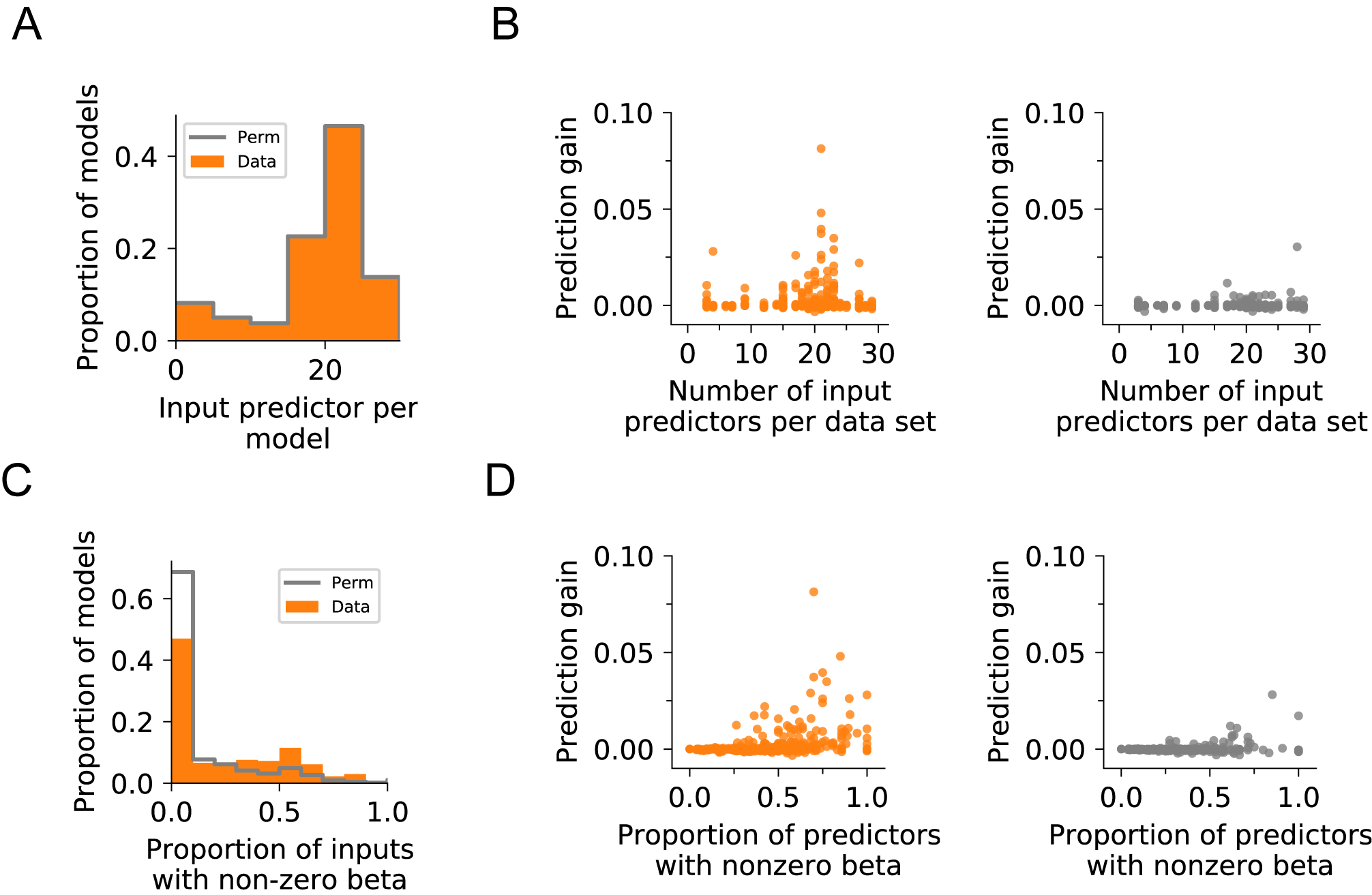

Supplement: S8 Fig — (A) Input predictor count for actual and permuted data sets. Wilcoxon rank sum test p = 1.0. (B) Prediction gain is not significantly correlated with the total number of input predictors used for prediction. Data: R2 = 0.00121, p = 0.384; permutation control: R2 = 0.00387, p = 0.118. (C) Models using actual data have higher proportions of predictors with nonzero β coefficients. Wilcoxon rank sum test p = 1.22 × 10^−16^. (D) Prediction gain is positively correlated with the proportion of input predictors with nonzero beta coefficients. This is found in both actual (left) and permuted (right) data sets. Data: R2 = 0.133, p = 3.10 × 10^−21^; permutation control: R2 = 0.0537, p = 0.3.80 × 10−9. Each point in the scatter represents a single fold of each model with 5 folds in total. All time points and models are shown. GLM, generalized linear model; PFC, prefrontal cortex. (TIF) [file pbio.3001393.s008.tif]
