# Supplementary material for: Prefrontal cortical activity predicts the occurrence of nonlocal hippocampal representations during spatial navigation
Source: PLoS Biol. 2021 Sep 16;19(9):e3001393. doi: 10.1371/journal.pbio.3001393 (PMC8494358; doi:10.1371/journal.pbio.3001393)
Supplement: Attachment — Submitted filename: Review_01_response_v05.pdf [file pbio.3001393.s010.pdf]

PBIOLOGY-D-21-01292R1

Prefrontal cortical activity predicts the extra-place field spiking of hippocampal place cells

Jai Y. Yu[1*] and Loren M. Frank[2]

Response to reviewers

Editorial and reviewer comments are in blue. Responses are in black.

We than the three reviewers for taking the time to carefully evaluate our work and for providing insightful and constructive comments. They have helped us improve the manuscript.

****

ETHICS REQUESTS

Please include the ID number of your experimental protocols approved by the University of California San Francisco Institutional Animal Care and Use Committee.

Experiments were conducted under protocol AN174991 approved by the University of California San Francisco Institutional Animal Care and Use Committee.

Please include the specific national or international regulations/guidelines to which your animal care and use protocol adhered. Please note that institutional or accreditation organization guidelines (such as AAALAC) do not meet this requirement.

The University of California San Francisco Institutional Animal Care and Use Committee meets all applicable federal, state, and University laws and regulations. USDA (#9199: #93-R-0440) and California Department of Fish & Wildlife (#1736).

We have added this to the Ethics Statement in the Methods section.

****

DATA SHARING REQUEST

As discussed above PLOS has a data policy, which requires that all data be made available without restriction: http://journals.plos.org/plosbiology/s/data-availability. For more information, please also see this editorial: http://dx.doi.org/10.1371/journal.pbio.1001797

We have made our raw data available without restriction.

Note that we do not require all raw data. Rather, we ask for all individual quantitative observations that underlie the data summarized in the figures and results of your paper. For an

example see here: http://www.plosbiology.org/article/info%3Adoi%2F10.1371%2Fjournal.pbio.1001908#s5

These data can be made available in one of the following forms:

1) Supplementary files (e.g., excel). Please ensure that all data files are uploaded as 'Supporting Information' and are invariably referred to (in the manuscript, figure legends, and the Description field when uploading your files) using the following format verbatim: S1 Data, S2 Data, etc. Multiple panels of a single or even several figures can be included as multiple sheets in one excel file that is saved using exactly the following convention: S1_Data.xlsx (using an underscore).

2) Deposition in a publicly available repository. Please also provide the accession code or a reviewer link so that we may view your data before publication.

Regardless of the method selected, please ensure that you provide the individual numerical values that underlie the summary data displayed in the following figure panels: Figures 1EF, 2B-D, 3BC, 4B, 5C, 6BD, S1, S2, S3AB, S4B-E, S5A-E, and S6BD.

NOTE: the numerical data provided should include all replicates AND the way in which the plotted mean and errors were derived (it should not present only the mean/average values).

We have included the required data for these and new figures.

***Please also ensure that each figure legend in your manuscript includes information on where the underlying data can be found and that your supplemental data file/s has/have a legend.

***Please ensure that your Data Statement in the submission system accurately describes where your data can be found.

****

REVIEWS:

Reviewer #1, Freyja Ólafsdóttir: Yu and colleagues aim to elucidate the function of place cell out-of-field activity. Traditionally, spikes occurring outside a place cell's (or any other neuron's) spatial receptive field is considered as noise. However, if this sporadic activity really reflects noise one would expect it to be uncoordinated in relation to other local and non-local cell activity. Yu and Frank find this is not the case. Rather out-of-field place cell activity (termed 'isolated spikes' by the authors) shows a strong phase preference along the movement-related theta rhythm, notably the phase preference differs for the isolated spikes compared to receptive-field spikes (termed 'adjacent spikes'). Further, adjacent co-activity patterns reflecting spatial field overlap are reactivated during isolated spikes. Finally, the study also shows that CA1 isolated spiking correlates with activity in the PFC and may be predicted by PFC activity.

Overall, I find the study's results interesting and I think the results may shed light on an under-studied activity phenomenon. I do have some questions regarding *what* this isolated activity reflects and require some methodological clarification regarding CA1-PFC coordination analyses. I outline my concerns below. All suggestions should be easily addressable by the authors and does not require the collection of more data.

1. My main questions concerns what this out-of-field activity represents. The authors suggest isolated activity could serve a sort of planning-like function serving decision making and goal directed behaviour. However, it would be useful to understand if isolated activity spikes could also be explained by other parameters.

a. For example, the authors show some isolated spikes seem to occur at locations where a place cell has a spatial receptive field, but for a different running direction. Could the authors show what proportion of isolated activity could perhaps be explained by such incomplete directional remapping?

This is an important point. With respect to the proportion of isolated spikes that occur in the opposite direction of adjacent spiking, we can estimate that based on the following plots. We determined the distribution of distance between isolated and adjacent spikes, and the distribution of spikes that cooccur in the same location but in the opposite direction of travel and we now show the results of these analyses in Fig. 2B and D. Based on Fig. 2B, we find ~44% of isolated spikes can be considered to occur within 1cm of adjacent spikes. Of those 44%, ~45% occur in the opposite direction (> 90°). Based on this conservative estimate, ~19% (.44 x .45) of isolated spikes occur in the opposite direction of travel.

b. Moreover, the animals are carrying out a highly dynamic task on the maze - i.e. the reward locations change every day/session. Could perhaps the isolated activity reflect the remnants of previous place fields or more generally instability of spatial representations on the task? Although this point is a bit tricky to answer, the authors could check if the occurrence of isolated spikes differs for cells that have ore more less spatial representations on the maze. Relatedly, if there are days/sessions where the reward contingency did not change for a protracted period, the authors could assess if in the more stable task conditions that occurrence of isolated spikes is reduced.

We thank the reviewer for raising this issue, as it led to an analyses, reported in Fig. 4, that provides clear support for the idea that these isolated spikes are related to learning of new task rules rather than spatial instability.

First, we note that our task is highly dynamic but the animals are well trained. Second, we carried out a set of analyses to examine the possibility that isolated spiking is related to instability in spatial representations, and our results do not provide support for that possibility.

We compared the rate of isolated spiking for the first and last 5 trials of each behavior epoch. We saw low level of performance in the first 5 trials, as measured by the probability of alternating between the rewarded wells, compared with the last 5 trials (Fig. 4D). The duration of the trials are similar, indicating the animals were familiar with the task structure and are trying different options to identify the correct reward locations (Fig. 4E).

We found significantly higher rates of isolated spiking during the first 5 trials versus the last (Fig. 4F). However, the same analysis for adjacent spiking does not show any difference (Fig. 4G). This adjacent spiking rate overall does not show any significant changes despite drastic changes in behavior. If new task rules from contingency changes affect spatial representation stability, we would have expected adjacent spiking to show similar changes between the first and last 5 trials of each session. The data supports isolated spiking reflecting planning during the learning of new reward locations than reflecting spatial representation instability, and we have added these findings to the manuscript.

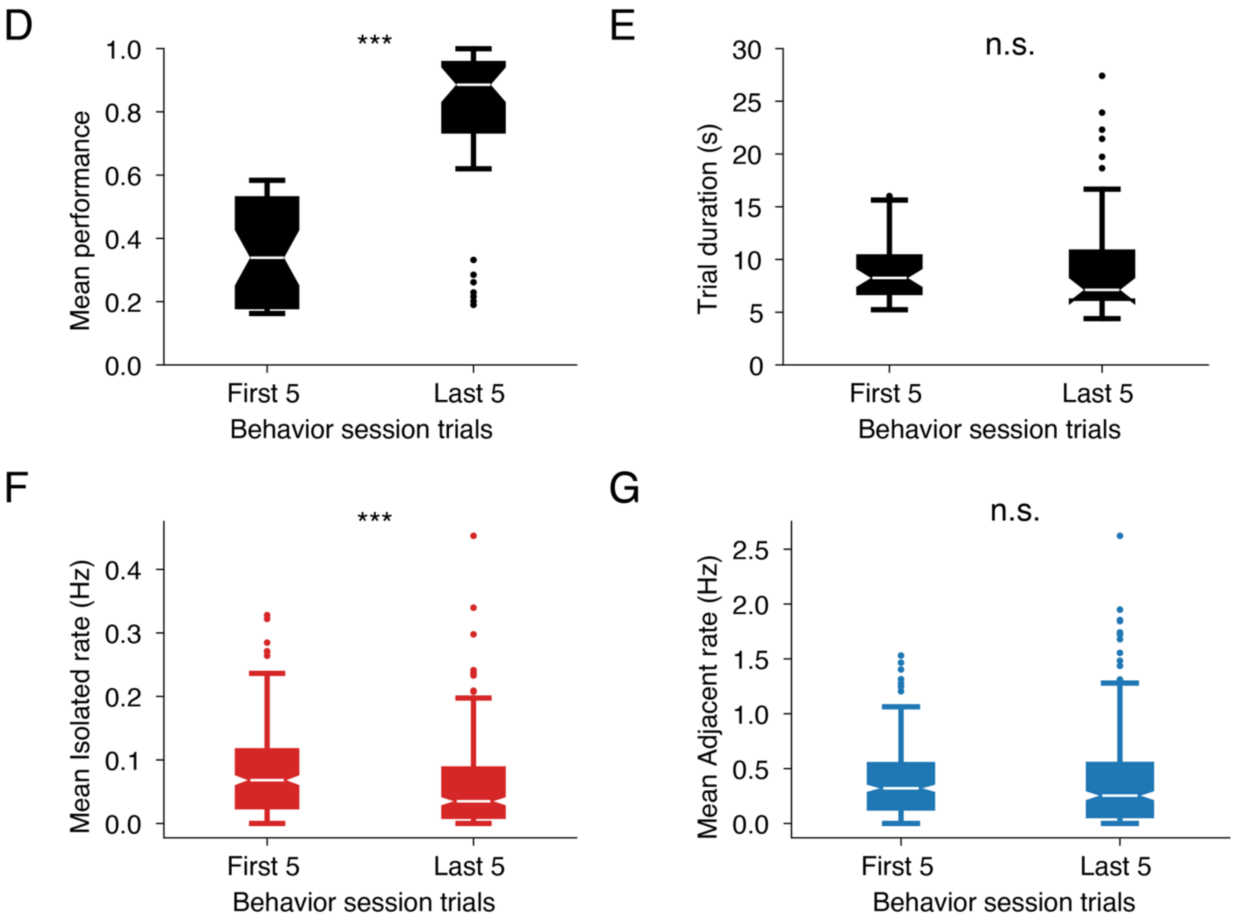

Figure 4. Spatial distribution and behavioral correlates of isolated and adjacent spiking.

D. Mean performance for the first and last 5 trials of each epoch across behavior sessions (n=38). Wilcoxon signed-rank test: $p=7.74\times10^{-8}$.

E. Median duration for the first and last 5 trials of each reward contingency across behavior sessions (n=38). Wilcoxon signed-rank test: p=0.89.

F. Mean isolated spiking rate for the first and last 5 trials of each reward contingency (n=247 cells). Wilcoxon signed-rank test: $p=9.79\times10^{-9}$.

G. Mean adjacent spiking rate for the first and last 5 trials of each reward contingency (n=247 cells). Wilcoxon signed-rank test: p=0.91.

Having found isolated spiking occurring more frequently during early trials, we wanted to ensure that this difference in time did not explain our observed PFC and CA1 prediction results. Specifically, once we match each isolated cycle with an adjacent cycle that occurred at a different time, we wanted to be sure that there was not an overall time difference between isolated and matched cycles. We there compared the time intervals between isolated cycles (median=48.4 min) versus the time intervals between isolated and matched cycles (median=49.5min) for each cell and the distributions were not significantly different at p=0.05 (n=159, p=0.067). We have mentioned this control analysis in the manuscript.

To address Reviewer 1's addition point that the rate of isolated spiking may be related to the cell's place field properties, we examined the correlation between key measure of spatial spread in adjacent spiking and the occurrence of isolated spiking and we have added these comparisons to Fig. S2. We used both the median and standard deviation of interspike distance between adjacent spikes to express their spread in space. We chose this method since our environment has a complex geometry. Such measure would capture the spread of adjacent spiking. We did not find a strong linear relationship between the extent of spatial spread of adjacent spikes and the rate of isolated spiking. Thus, we do not find that isolated spiking has a simple relationship with place cells that have sparse place field spiking.

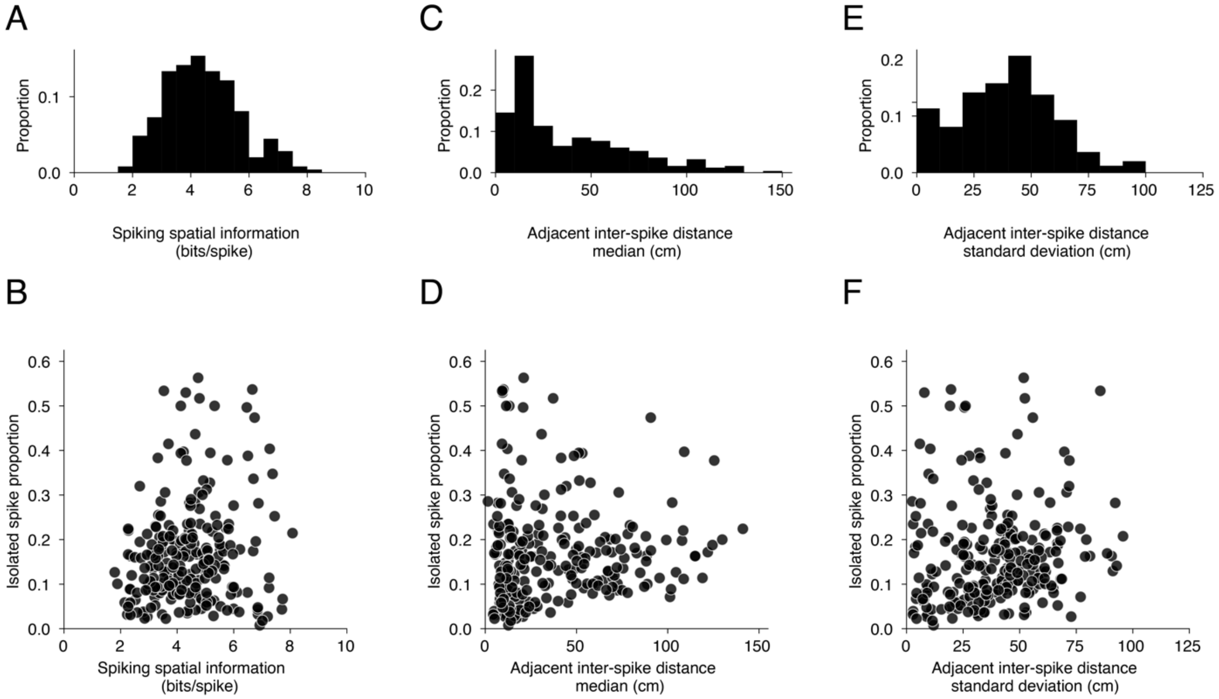

Figure S 2. Spatial distribution of adjacent spiking is not correlated with the proportion of isolated spiking.

A. Distribution of spatial information. Median: 4.35 bits/spike.
B. The proportion of isolated spikes is poorly correlated with spiking spatial information (n=247). R^2=0.0246 p=0.014.
C. Distribution of the median distance between adjacent spikes. Median: 24.04 cm.
D. The proportion of isolated spikes is not significantly correlated with the median distance between adjacent spikes (n=247). R^2=0.0081 p=0.16.
E. Distribution of the standard deviation of distance between adjacent spikes. Median: 40.18cm.
F. The proportion of isolated spikes is not significantly correlated with the standard deviation of distance between adjacent spikes (n=247). R^2=0.0089 p=0.14.

This points speaks partly to recent findings showing artificial remapping in CA1 cells is not random but can rather be predicted by residual (i.e. isolated) activity prior to the induction of remapping, suggesting the existence of 'dormant' place fields. Thus, could the isolated activity the authors describe here reflect such dormant firing fields?

As the reviewer points out, recent findings indicate place fields is more likely to be induced artificially at locations where residual spiking occurs (McKenzie et al., 2021). If the isolated spikes were best understood as dormant place fields, we would expect the spikes to occur throughout the behavioral session, reflecting occasions when the spatial inputs became suprathrehold. The analyses above indicate that this is not the case; instead, isolated spiking is more frequent at the start of new epochs. The subset of isolated spikes occurring in the opposite direction of travel to a cell's place field could reflect the suppression of place field firing, however, the function of these spikes in remains to be determined.

2. Fig 5B shows examples of isolated and control activity from CA1 cells, however I am unsure how these plots are made. Does each row represent a trial (or a run) through a particular location? Is the location the same across trials, or is each row showing an example of isolated activity occurring in different places? Moreover, some of these examples show clear clustering of CA1 spikes around cycle 0, how can this be? The definition of isolated activity is that CA1 spikes are occurring nearly on their own, so how can they cluster?

Here we failed to clearly explain the methods. The CA1 isolated spikes in this figure shows time intervals aligned to the theta cycle in which they occurred in. These time intervals are not at the same location since isolated spiking occurs at different places on the maze. We selectively aligned the cycles for this analysis. The rationale is if there are systematic changes in PFC activity around these events, we can detect these changes be aligning all the CA1 isolated events in time.

The perceived clustering is due to the way we visualize the spiking events. Since many isolated spiking events are shown in the panel, we had to make each spike visible as a circle, otherwise they will be too small to see. It is possible to have isolated spiking on two consecutive theta cycles as isolated spiking is defined as separation to its closest 3 neighboring theta cycles (Fig. S1), so sparse spiking in time but across two cycle but still separated from the majority of adjacent spiking can still be considered isolated.

We have clarified this point in :
Line 121 - This method identifies spiking on individual theta cycles and also spiking on a small number of nearby cycles that are nevertheless isolated from periods of adjacent spiking.

Line 333 - Although isolated spiking does not occur at specific locations, we can use the times of isolated spiking events as reference points to look for coordination with PFC activity.

3. Finally, I have a few related questions about the PFC-CA1 interaction analyses. Namely, fig5b seems to show some PFC cells decrease their activity in relation to the occurrence of an isolated CA1 spike while others increase, is this the case? Could the authors quantify the number of PFC cells which show down-regulation vs up-regulation? Moreover, fig5c shows PFC activity changes at different time points leading up to the occurrence of an isolated CA1 spike - does the activity change increase as an closer to the time of an isolated spike (i.e. between theta cycles -4- 1 vs cycles 0-1)? Similarly, does the PFC prediction strength significantly increase at time intervals closer to cycle 0?

Reviewer 1 is correct in concluding PFC cells can increase or decrease spiking relative to isolated CA1 spiking. However, this relationship is unique to pairs of PFC-CA1 cells. One PFC cell may not increase their activity to isolated spiking from all CA1 cells. We have added the distribution of raw z scores in Fig. S 7.

This analysis required PFC-CA1 cell pairs where the PFC cell had enough spiking across the time bins to calculate a meaningful difference between the isolated and matched cycles. Our original analysis did not select for this and included all pairs. We thus restricted the data to PFC-CA1 pairs where the PFC cell had higher occurrence of spiking, which we define as spiking in at least 25% of isolated or matched cycles.

First, we calculated the proportion of PFC-CA1 cell pairs with a significant firing rate difference based on a p<0.05 from the permutation test. We would expect a chance value of 5% of data points. We observed higher proportions in the data than chance but chance levels in the permuted dataset.

	**Data**		**Permutation control**	
Time window	Permutation test, proportion <0.05	Binomial test vs 5% expected (p-value)	Permutation test, proportion <0.05	Binomial test vs 5% expected (p-value)
-12 to -8	0.094	1.38×10^{-10}	0.046	0.558
-8 to -4	0.097	7.68×10^{-12}	0.049	1.0
-4 to 0	0.125	4.26×10^{-25}	0.056	0.298
0	0.121	1.90×10^{-18}	0.057	0.301

Next, we highlighted and included the proportion of PFC-CA1 cell pairs where the PFC firing rate either decreased or increased by at least 2-z. Although we see more PFC firing increases, we are hesitant to draw conclusions about whether there were differences in the proportions of each for the whole population. While these figures suggest that there may be more positive than negative changes, this can be caused by the fact that rates can only go down to 0, whereas increases are, theoretically, boundless. Given this complexity, we do not feel comfortable coming to a conclusion about whether there are more increases or decreases.

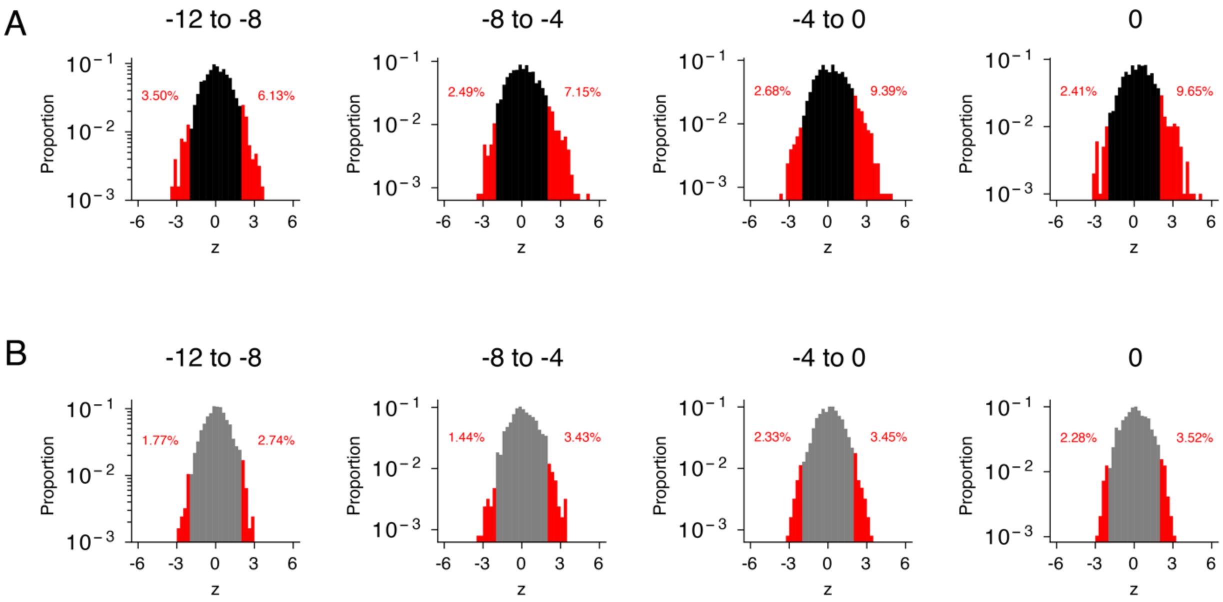

Figure S 7. PFC firing rate change in time bins relative to isolated CA1 spiking.

A. Distribution of PFC firing rate change (z) for time windows before and during CA1 isolated spiking in Fig. 6C. Bins above and below 2D are colored red and the proportion of the distribution are printed in red. Only PFC-CA1 cell pairs where PFC spiking occurs in at least 25% of isolated or matched cycles are included. This selects for PFC cells with higher spiking rates.
B. Distribution of permuted PFC firing rate change (z) for time windows before and during CA1 isolated spiking in Fig. 6C.

To address Reviewer 1's question about whether PFC activity change increases over time relative to the occurrence of CA1 isolated spiking, we performed a linear regression on the magnitude of change and the time bins. The R-slope is positive but very small, with a significant p-value. This relationship is not observed for the permutation control. While we are confident there is a positive slope, it is small and we would want to avoid making a strong statement about the change over the time bins. We have therefore left this out of the manuscript, although we are happy to reconsider if the reviewer feels strongly about this.

	Data	Permutation control
**R**	0.036	0.0075
**p**	0.000026	0.37
**n**	14058	14058

For the prediction significance across time bins, we also performed a linear regression. Again we see a very weak but significant positive slope only in the data group indicating the strength of the prediction increases over the time bins. We have added this result in the manuscript (line 455).

	Data	Permutation control
**R**	0.11	0.0005
**p**	0.0073	0.99
**n**	632	632

Reviewer #2: This paper reports an analysis of the out-of-field sparse spiking activity of hippocampal place cells. The authors examined such firing during locomotion as rats were running a maze in which they had to commute back and forth between several goal locations. They showed that isolated spikes recorded from place cells were coherently organized and were phase coupled to theta oscillations in a way different from the phase coupling observed when the cells fire in the place field. Furthermore the authors demonstrate isolated spikes in hippocampal cells are driven by prefrontal cortical activity. They conclude that out-of-field isolated firing is not noise but a signature of distributed and coherent information processing in the brain.
This is an excellent paper that reports an original and very important finding. I have no major concerns. The question is relevant, the analyses are well done and the results are clear.
I have only a few minor suggestions.

1) Although the present study focused on isolated spikes during locomotion and theta state, other studies have reported the existence of such out-of-field sparse firing during quiet alertness associated with theta state while the rat was waiting at a goal location (Hok et al., J. Neuroci. 1987). Some of the observations made in the Hok et al's paper were similar to those made here and it would be useful to cite this older study just to emphasize that isolated spikes might not occur only during locomotion.

We have revised the text (line 40, reference 17) to reference this important study.

2) In a similar vein, it is worth mentioning the paper by Hasz and Redish (Hippocampus 2020) reporting a firing relationship between the medial prefrontal cortex and the hippocampus. This work shows that medial prefrontal activity influences hippocampal activity in a way that is consistent with the present observations.

We have revised the text (reference 77) to include this study.

3) It would be helpful to include some additional information about the key spatial features of the place cell sample used here, just to convince the reader that the analysis was done on "good" place cells and not a noisy sample.

Reviewer 2 suggestion is related to Reviewer 1's comment about whether isolated spiking may reflect "noisy" place cells with diffuse place fields.

We have now included data on the spatial information and spatial spread of spiking for our cell population (Fig. S 2). We also recomputed key analysis (Fig. 2 B-D and Fig. 3 B-C) by only including cells with at least 50 adjacent and 5 isolated spikes. The results of the comparisons between the two types of spiking remain the same. Thus, our findings do not indicate isolated spiking results from a "noisy" sample of place cells.

4) Really minor:

* Line 144, add "and" before "its"

Corrected.

* Line 151, "place cells" instead of "place cell"

Corrected.

* Line 241, "similar" instead of "similarity"

Corrected.

* Line 383, it is unclear what "outside of a place cell's place and movement direction field" means

We removed "movement field".

* Line 392, delete "during"

Deleted.

Reviewer #3, Zaneta Navratilova: This paper shows that hippocampal CA1 spikes that occur outside of "place fields" reflect coherent activity within the hippocampus, and are coordinated with activity in medial prefrontal cortex, showing that they cannot be characterized as local "noise." This important insight is shown with a well-designed and clear series of analyses.
I have just a few comments to improve the paper.

* The main text of the paper is well argued and clear. However, the figure legends are sometimes incomplete, and not clear enough for a non-expert reader.

We have improved the readability and the missing legends (previously Fig. 6, now Fig. 7).

* Some supplementary figures (in particular figures S2 and S4) are very important controls, necessary to the conclusions of the paper, and the authors should consider including them as main figures.

This is a very useful suggestion and improves the overall presentation of the data. We have rearranged the panels so that the relevant parts of the figure are now in the main figure. (compare previous Figs. S4 and new Fig. 4). The new Fig. 4 will address spatial and behavioral correlated of isolated spiking, thus combining both Reviewer 1 and 3's comments.

* Figure 5 compares PFC activity before and during theta cycles that include isolated spikes to similar theta cycles with no isolated spikes. Why not also compare the activity after isolated spikes? It is clear based on figure 6 that there is also a difference in PFC activity following isolated spikes, and it is not evident why the authors exclude this period only in figure 5.

This is great suggestion to improve the completeness of the manuscript and we have added the equivalent for the post-isolated PFC activity difference.

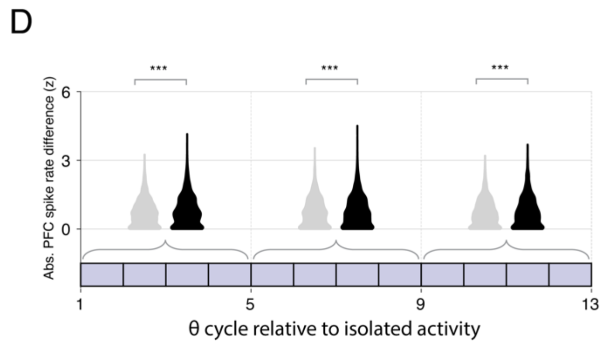

Figure 6. PFC activity is coordinated with hippocampal isolated activity.

D. Violin plots and quantification of spike rate differences between control and actual intervals for PFC-CA1 cell pairs (n=2892) in time windows post CA1 isolated activity. The Wilcoxon signed-rank test (*** p<0.001, ** p< 0.01) was used to compare the original and permuted groups: $p=9.5\times10^{-7}$, 1.8×10^{-3} and 2.1×10^{-3} for each group respectively.

* In contextualizing this paper with previous studies in the literature, the authors miss a few relevant findings: in addition to different trajectories or future locations, non-local place cell spiking can also reflect different environments (Jezek et al., Nature, 2011) or reference frames, which are sometimes represented in alternating theta cycles, or simultaneously in the late phases of theta (Kelemen and Fenton, Plos Biology, 2010). In addition, spiking during travel in the non-preferred direction has been shown to occur more commonly and at higher rates during early stages of learning a specific trajectory through an environment (Navratilova et al., Frontiers in Neural Circuits, 2012). These findings suggest that "non-local" spiking can occur in different amounts, not just as isolated spikes. A discussion of whether the authors believe these are different phenomena (e.g. existing on a gradient of a single isolated spike to multiple adjacent or alternating theta cycles of non-local spikes), or if isolated spiking is a separate phenomenon, would be welcome.

We thank the reviewer for pointing these relevant studies out and have corrected the omission. We had added the additional references to the discussion (references 64, 65 and 10).

With respect to the change in spiking in the non-preferred direction of travel, we want to present some differences between our and the previous study by the reviewer. Our recordings were done after the animal is familiar with the environment and has learned the contingency switch task (>10 days of exposure). In such environments, place representations would have become more stable. Although we see higher rates of isolated spiking during the early trials of a behavior epoch this is likely to reflect isolated spiking rather than the establishment of directionality observe during early learning.

Minor edits:

* Figure S4 legend is grammatically unclear; should read: "Animal location, movement correlates and hippocampal network spectral signatures around excluded, adjacent and isolated spiking activity."

We have changed the legend. Parts of the figure are now in Fig. 4.

* Typo on line 241: should read: "very similar"

Corrected.
