# Supplementary material for: Prefrontal cortical activity predicts the occurrence of nonlocal hippocampal representations during spatial navigation
Source: PLoS Biol. 2021 Sep 16;19(9):e3001393. doi: 10.1371/journal.pbio.3001393 (PMC8494358; doi:10.1371/journal.pbio.3001393)
Supplement: Attachment — Submitted filename: PlosBiology_revision2_responsetoeditor.pdf [file pbio.3001393.s011.pdf]

PBIOLOGY-D-21-01292R1

Prefrontal cortical activity predicts the extra-place field spiking of hippocampal place cells

Jai Y. Yu[1*] and Loren M. Frank[2]

Response to the editor

Thank you for submitting your revised Research Article entitled "Prefrontal cortical activity predicts the extra-place field spiking of hippocampal place cells" for publication in PLOS Biology. I have now discussed your revision with other staff editors and with the Academic Editor as well. I am pleased to tell you that we will probably accept this manuscript for publication, provided you update your supporting data folder to provide the data for figures 4E and S3AB.

We have included the missing data for figures 4E and S3A and B. We have updated the format of the data files and have uploaded a new zipped file containing data for all figures. Please use this new zip file.

In addition, we would like you to consider changing the title to make it more accessible to a broader audience. We thought about the following but would be happy to discuss an alternative if you feel our suggestion misrepresents your findings or is inaccurate:

"Spiking of hippocampal place cells outside the place field reflects non-local spatial representations during spatial navigation."

Thank you for suggesting that we change our title to appeal to a broader audience. We would like to suggest the following: ***"Here but thinking of there: prefrontal cortical activity predicts the occurrence of non-local hippocampal representations during spatial navigation"***.

The strength and novelty of our work lies in demonstrating the coordination between prefrontal cortex and the hippocampus during transient non-local representations. The ability for prefrontal cortical activity to predict these events is key to understanding how non-local representations in the hippocampus may arise. We feel it is important to include this in the title. We are open to other suggestions for a more appropriate title that will appeal to PLOS Biology readers.
